# Preparation of Porous Ti/RuO_2_-IrO_2_@Pt, Ti/RuO_2_-TiO_2_@Pt and Ti/Y_2_O_3_-RuO_2_-TiO_2_@Pt Anodes for Efficient Electrocatalytic Decomposition of Tetracycline

**DOI:** 10.3390/molecules28052189

**Published:** 2023-02-27

**Authors:** Yunqing Zhu, Bingqing Li, Yongming Wang, Tian Wang

**Affiliations:** School of Environmental Science and Engineering, Shaanxi University of Science and Technology, Xi’an 710021, China

**Keywords:** electrocatalytic oxidation, platinum, porous electrode, tetracycline hydrochloride, Ti/Y_2_O_3_-RuO_2_-TiO_2_@Pt anode

## Abstract

Electrocatalytic oxidation (ECO) has attracted attention because of its high efficiency and environmental friendliness in water treatment. The preparation of anodes with high catalytic activity and long service lifetimes is a core part of electrocatalytic oxidation technology. Here, porous Ti/RuO_2_-IrO_2_@Pt, Ti/RuO_2_-TiO_2_@Pt, and Ti/Y_2_O_3_-RuO_2_-TiO_2_@Pt anodes were fabricated by means of modified micro-emulsion and vacuum impregnation methods with high porosity titanium plates as substrates. The scanning electron microscopy (SEM) images showed that RuO_2_-IrO_2_@Pt, RuO_2_-TiO_2_@Pt, and Y_2_O_3_-RuO_2_-TiO_2_@Pt nanoparticles were coated on the inner surface of the as-prepared anodes to form the active layer. Electrochemical analysis revealed that the high porosity substrate could result in a large electrochemically active area, and a long service life (60 h at 2 A cm^−2^ current density, 1 mol L^−1^ H_2_SO_4_ as the electrolyte, and 40 °C). The degradation experiments conducted on tetracycline hydrochloride (TC) showed that the porous Ti/Y_2_O_3_-RuO_2_-TiO_2_@Pt had the highest degradation efficiency for tetracycline, reaching 100% removal in 10 min with the lowest energy consumption of 167 kWh kg^−1^ TOC. The reaction was consistent with the pseudo-primary kinetics results with a k value of 0.5480 mol L^−1^ s^−1^, which was 16 times higher than that of the commercial Ti/RuO_2_-IrO_2_ electrode. The fluorospectrophotometry studies verified that the degradation and mineralization of tetracycline were mainly ascribed to the •OH generated in the electrocatalytic oxidation process. This study thus presents a series of alternative anodes for future industrial wastewater treatment.

## 1. Introduction

Tetracycline hydrochloride (TC) is a typical broad-spectrum antibiotic that is widely used in treating infectious diseases of humans and livestock [1,2]. The production and consumption amount of tetracycline are more than several tens of thousands of tons per year, which makes it one of the most widely detected antibiotics in the world at the levels of mg L^−1^ in soil and ng L^−1^-μg L^−1^ in natural water [3,4]. Furthermore, tetracycline has persistently existed in the aquatic ecosystem, and it is difficult to metabolize in the microbial system, which represents a long-term threat to human health and the ecological environment [5,6,7]. Therefore, dealing with the tetracycline pollution problem has become an increasingly prominent issue. Several studies have been carried out in the past decades to develop efficient methods for tetracycline degradation, such as adsorption [8,9,10], membrane separation [11], chemical oxidation [12], etc.

Recently, advanced oxidation technology (AOT) (e.g., Fenton oxidation, ozonation, photocatalysis, and electrocatalytic oxidation) has been applied as a primary chemical oxidation method in many water treatment plants due to its strong oxidation capability [10]. Among the available AOTs, electrocatalytic oxidation (ECO) is recognized as a highly efficient, environment-friendly and adaptable technology [13]. In the ECO process, the effective degradation of refractory organic contaminants (ROCs) is mainly ascribed to the •OH generated and simultaneously to reactive chlorine species [14], which substantially enhanced the degradation of ROCs [15,16]. However, to develop a practically applicable ECO technology, the development of highly active anode materials with a long service life is the key to competitiveness [17]. In recent decades, planar electrodes (e.g., boron-doped diamond (BDD), SnO_2_-Sb, PbO_2_, and RuO_2_-IrO_2_-coated Ti) have been intensively investigated [17,18,19,20,21]. Ti/BDD electrodes, as demonstrated by Yang et al., can achieve a tetracycline degradation of 100% at a high current density of 150 mA cm^−2^ after 2 h of reaction [22]. Ti/SnO_2_-Sb anodes fabricated by means of the electro-deposition method can obtain 97% tetracycline degradation at 20 mA cm^−2^ [23]. Additionally, the Ti_4_O_7_ anode was also found to reach a tetracycline degradation level of 90% at 3 mA cm^−2^ in 0.1 M Na_2_SO_4_ electrolyte [24]. Although the traditional planar anodes have well-recognized catalytic activity, their poor electrochemically active surface area (ECSA) and mass transfer performance limit their further application. In our previous work, a series of mesoporous structures in the active layer were constructed, which resulted in not only an enhanced catalytic activity, but also an extended service life [25,26]. Pang et al. developed trace Ti^3+^- and N-codoped TiO_2_ nanotube array (NTA) anodes, which also achieved improved activity in terms of tetracycline decomposition compared with its available counterparts [27]. Meanwhile, Chen et al. coated TiO_2_ NTA with a SnO_2_-Sb layer and found that the oxygen evolution potential and •OH production were both improved, accompanied by an improvement in its conversion service lifetime [28]. Furthermore, a porous Ti_4_O_7_ membrane electrode prepared via a simple one-step spark plasma sintering method was reported by Lin et al., which led to a high oxygen evolution potential (OEP), a larger electrochemically active area, and a faster inter-facial mass transfer rate [29]. All these works demonstrated that the use of a porous structure, whether in the active layer or in bulk, apparently increases the ECSA and mass transfer performance, and leads to enhanced catalytic activity and a longer service life [30]. However, although previous studies have provided inspiration for the further development of effective anodes, exploiting a practical applicable porous anode remains a challenge and has rarely been studied until now.

Herein, porous Ti/RuO_2_-IrO_2_@Pt, Ti/RuO_2_-TiO_2_@Pt, and Ti/Y_2_O_3_-RuO_2_-TiO_2_@Pt electrodes were prepared using modified microemulsion and vacuum impregnation methods with commercial porous titanium plate used as a substrate. The physicochemical and electrochemical properties of the as-prepared electrodes were characterized. The electrocatalytic oxidation efficiency of the electrodes with regard to tetracycline was evaluated systematically, and the important contributing factors (e.g., the electrolyte, concentration of the electrolyte, current density, initial pH, and initial concentration of tetracycline) were investigated. Additionally, the concentration of ·OH was tested to understand the mechanism of tetracycline degradation, and an accelerated lifetime test was performed to evaluate the stability of the as-prepared electrodes. This study may provide a series of alternative anodes that can be used practically in ECO projects for wastewater treatment.

## 2. Results and Discussion

### 2.1. Morphological and Structural Characterization

**Figure 1:**
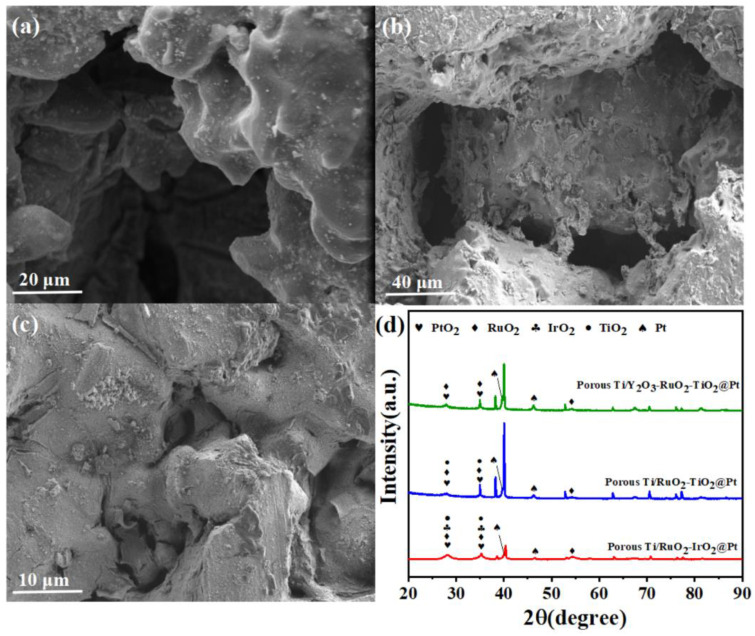
Scanning electron microscope images of (**a**) porous Ti/RuO_2_-IrO_2_@Pt, (**b**) porous Ti/RuO_2_-TiO_2_@Pt, and (**c**) porous Ti/Y_2_O_3_-RuO_2_-TiO_2_@Pt electrodes. (**d**) X-ray diffractometer patterns.

The morphology of the prepared porous Ti/RuO_2_-IrO_2_@Pt, Ti/RuO_2_-TiO_2_@Pt, and Ti/Y_2_O_3_-RuO_2_-TiO_2_@Pt anodes were characterized via SEM and EDS. As shown in Figure 1a–c, the surface structure was apparently changed after coating with RuO_2_-IrO_2_@Pt, RuO_2_-TiO_2_@Pt, and Y_2_O_3_-RuO_2_-TiO_2_@Pt composites. The precursor solution was filled in the inner pores of the porous Ti plate and coated on the surface of Ti particles. The pore structure was analyzed by means of the injection of mercury, as shown in Figure S2 and Table S1, and the mercury intrusion pore size distribution further indicated that the inner pores were filled, since the average pore size was reduced from 24.47 μm to 10.89 μm, 14.84 μm, and 12.64 μm, respectively, after coating with the RuO_2_-IrO_2_@Pt, RuO_2_-TiO_2_@Pt, and Y_2_O_3_-RuO_2_-TiO_2_@Pt composites. In contrast, the total pore capacity and total pore area were increased after coating, which can probably be ascribed to the microstructure of the composite coating layer. Additionally, compared with Ti/RuO_2_-TiO_2_@Pt, Ti/RuO_2_-IrO_2_@Pt, and Ti/Y_2_O_3_-RuO_2_-TiO_2_@Pt, the anodes showed a much smoother surface with very few defects, which may lead to a much more stable performance in the ECO process [31,32,33]. The EDS mapping (Figure S3) showed that for the prepared porous Ti/RuO_2_-IrO_2_@Pt, Ti/RuO_2_-TiO_2_@Pt, and Ti/Y_2_O_3_-RuO_2_-TiO_2_@Pt anodes, the elements of Ru, Pt, Ti, and Y were uniformly distributed on the electrode surface. The chemical structure of the anodes was characterized using XRD spectroscopy (Figure 1d). According to the Joint Committee on Powder Diffraction Standards (JCPDS) card, the main diffraction peaks identified near 28°, 35°, and 54° were ascribed to RuO_2_ (JCPDS 88-0323)-based mixed oxides with IrO_2_ (JCPDS 86-0330), PtO_2_ (JCPDS 75-1059), or TiO_2_ (JCPDS 88-1175). The peak that appeared at 46.3° corresponded to Pt^0^ (JCPDS 04-0802). Identified using the microemulsion method, Pt^0^ could be held in the composite, and this probably led to an enhancement in the electron transfer capability and resulted in efficient performance in the ECO process [34,35,36,37].

### 2.2. Electrochemical Performance Analysis

**Figure 2:**
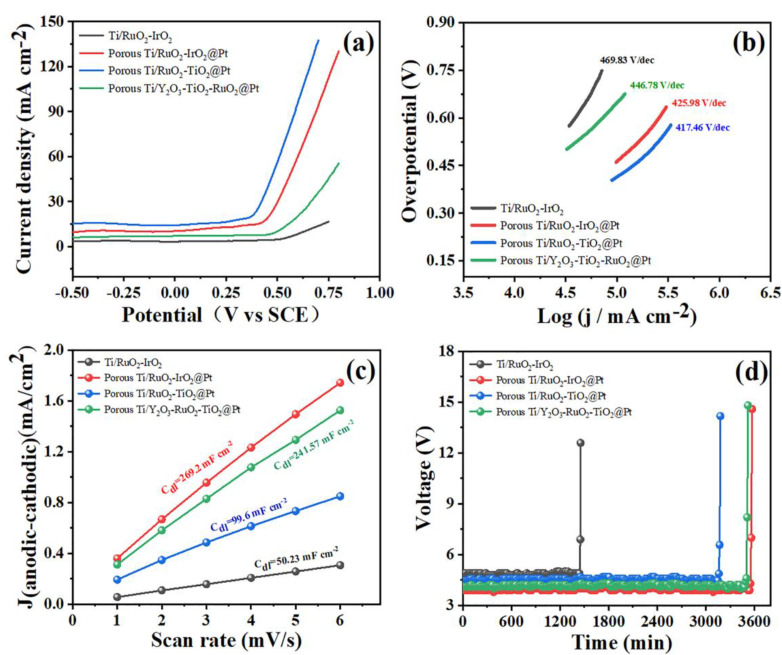
Electrochemical characterization of porous Ti/RuO_2_-IrO_2_@Pt, porous Ti/RuO_2_-TiO_2_@Pt, and porous Ti/Y_2_O_3_-RuO_2_-TiO_2_@Pt electrodes. (**a**) LSV and (**b**) related Tafel plots of Ti/RuO_2_-IrO_2_, porous Ti/RuO_2_-IrO_2_@Pt, porous Ti/RuO_2_-TiO_2_@Pt, and porous Ti/Y_2_O_3_-RuO_2_-TiO_2_@Pt. Experimental conditions: scan rate of 50 mV s^−1^ and 1.0 M KOH. (**c**) Evaluation of C_dl_ values, plotting J vs. scan rate. (**d**) Accelerated life testing results. Experimental conditions: current density of 2 A cm^−2^ and 1.0 M H_2_SO_4_.

The electrochemical performance of each anode was tested via the linear sweep voltammetry (LSV) technique using a three-electrode system with 1.0 M KOH electrolytes [38,39,40]. The collected LSV polarization curves are shown in Figure 2a. We discovered that the addition of Pt in the composites could enhance its activity in the oxygen evolution reaction (OER), which resulted in lower potentials of ~0.38 V for Ti/RuO_2_-TiO_2_@Pt, ~0.44 V for Ti/RuO_2_-IrO_2_@Pt, and ~0.49 V for Ti/Y_2_O_3_-RuO_2_-TiO_2_@Pt (vs. SCE), respectively, compared with that of the commercial Ti/RuO_2_-IrO_2_ electrode. In addition, the kinetics of the anodes for OER were investigated through the Tafel plot (Figure 2b). The Tafel slope of commercial Ti/RuO_2_-IrO_2_ was 469.83 mV dec^−1^, whereas those of the porous Ti/RuO_2_-TiO_2_@Pt, Ti/RuO_2_-IrO_2_@Pt, and Ti/Y_2_O_3_-RuO_2_-TiO_2_@Pt were 417.46, 425.98, and 446.78 mV dec^−1^, respectively. There was not much of a difference among the Tafel slope values of the electrodes, which could be due to the fact that the main component of all the electrodes was RuO_2_, and this had the main influence on the kinetics of the anodes for OER. ECSA analysis was performed to evaluate the catalytic activity of the prepared electrodes in the ECO reaction. Cyclic voltammetry (CV) curves were obtained at scan rates from 10 to 60 mV s^−1^ in a non-Faraday section for the calculation of double-layer capacitance (C_dl_) values [41,42]. The C_dl_ values had a positive correlation with the ECSA results of the electrodes. As shown in Figure 2c, the calculated C_dl_ value of porous Ti/RuO_2_-IrO_2_@Pt was 269.2 mF cm^−2^, which was 5.4 times higher than that of commercial Ti/RuO_2_-IrO_2_ electrodes. This result indicated that the porous Ti substrate could substantially increase the ECSA of the electrodes, which led to an increase in the number of revelational active sites and boosted their electrical catalytic activity in the ECO reactions. In addition, accelerated life testing was conducted in 1 M H_2_SO_4_ solution at 2 A cm^−2^ to assess the stability of the electrodes [43]. We observed that the commercial Ti/RuO_2_-IrO_2_ electrode only held for 1475 min, whereas the porous Ti/Y_2_O_3_-RuO_2_-TiO_2_@Pt and Ti/RuO_2_-IrO_2_@Pt electrodes lasted 3576 and 3588 min, respectively, which were twice as high as that of the commercial Ti/RuO_2_-IrO_2_ electrode (Figure 2d). This shows that the porous electrode surface structure was dense and the electrocatalytic layer formed was more stable, which effectively prevented the corrosion of the electrolyte and enhanced the service life of the electrode.

### 2.3. Electrocatalytic Degradation of Tetracycline

#### 2.3.1. Effect of Electrolyte Type and Concentration

**Figure 3:**
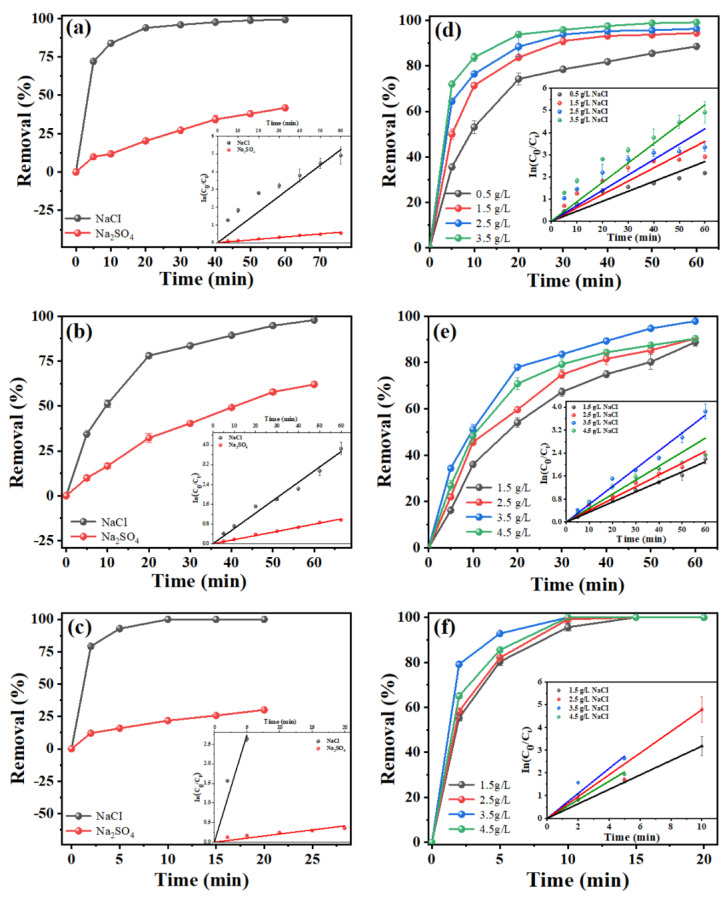
Effect of electrolyte type on degradation efficiencies. (**a**) Porous Ti/RuO_2_-IrO_2_@Pt, (**b**) porous Ti/RuO_2_-TiO_2_@Pt, (**c**) porous Ti/Y_2_O_3_-RuO_2_-TiO_2_@Pt electrodes. Experimental conditions: current density of 20 mA cm^−2^, initial TC concentration of 50 mg L^−1^, and electrolyte concentration of 3.5 g L^−1^, pH = 7. Effect of electrolyte concentration on degradation efficiency. (**d**) Porous Ti/RuO_2_-IrO_2_@Pt, (**e**) porous Ti/RuO_2_-TiO_2_@Pt, (**f**) porous Ti/Y_2_O_3_-RuO_2_-TiO_2_@Pt electrodes. Experimental conditions: current density of 20 mA cm^−2^ and TC initial concentration of 50 mg L^−1^, pH = 7. Inserts show the corresponding kinetics analysis.

Testing of the degradation of tetracycline with NaCl and Na_2_SO_4_ electrolyte solutions was conducted to evaluate the effect of electrolytes on the electrocatalytic oxidation of tetracycline [44]. As shown in Figure 3, for all the electrodes, the tetracycline degradation efficiency of NaCl as an electrolyte was much higher than that of Na_2_SO_4_. This can probably be ascribed to the fact that Cl^−^ ions in the solution could be oxidized, generating •Cl, HClO, and other reactive chlorine species, which could further react with tetracycline molecules or by-products to enhance the tetracycline degradation efficiency [45]. In addition, the effect of the NaCl concentration on the degradation of tetracycline was also studied (Figure 3). As presented in the inserts of Figure 3d–f, the kinetics under different NaCl concentrations were consistent with pseudo-first-order kinetics. For porous Ti/Y_2_O_3_-RuO_2_-TiO_2_@Pt, the calculated k value increased with increasing NaCl concentration and reached the maximum value of 0.55 min^−1^ at an NaCl concentration of 3.5 g L^−1^. Meanwhile, 38.2% TOC removal was achieved in a 20 min reaction time, accompanied by an EC of 167 kWh kg^−1^ TOC (Figure S5). For porous Ti/RuO_2_-TiO_2_@Pt and Ti/RuO_2_-IrO_2_@Pt, the kinetics constants reached the maximum value of 0.06 and 0.09 min^−1^ at a NaCl concentration of 3.5 g L^−1^, respectively. The TOC removal reached 25.9% and 48.1% in 60 min of reaction for porous Ti/RuO_2_-TiO_2_@Pt and Ti/RuO_2_-IrO_2_@Pt, respectively (Figure S5). The positive effect of the NaCl electrolyte was due to the oxidation of Cl^−1^ to active chlorine substances on the anode surface during the ECO process. Similar results were also reported in previous studies on the degradation of triclosan [46,47], rhodamine B [47,48], and enrofloxacin [47,49].

#### 2.3.2. Effect of Current Density

**Figure 4:**
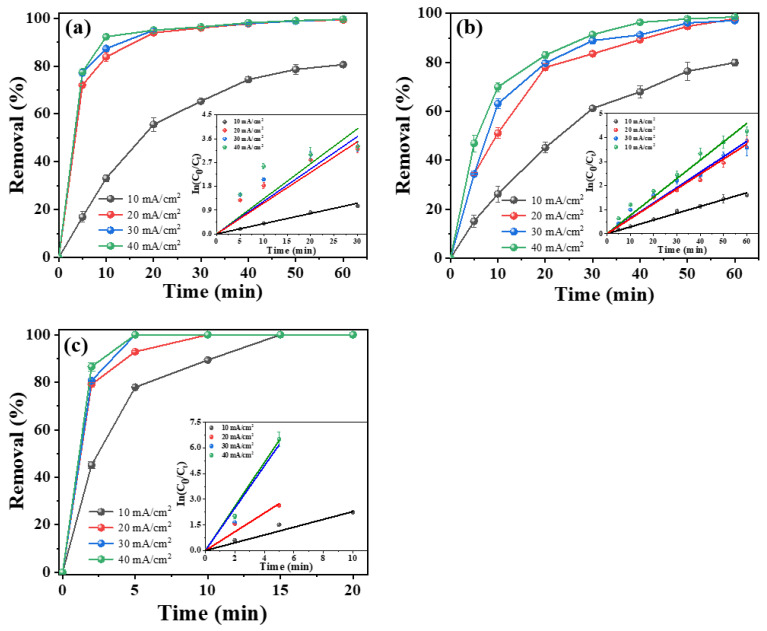
Effect of current density on degradation efficiency. (**a**) Porous Ti/RuO_2_-IrO_2_@Pt, (**b**) porous Ti/RuO_2_-TiO_2_@Pt, (**c**) porous Ti/Y_2_O_3_-RuO_2_-TiO_2_@Pt electrodes. Experimental conditions: initial TC concentration of 50 mg L^−1^ and electrolyte concentration of 3.5 g L^−1^ NaCI, pH = 7. Inserts show the corresponding kinetics analysis.

In the ECO process, the current density applied in the system is a crucial parameter for the generation of hydroxyl radicals. An increase in the current density can accelerate the yield of •OH and enhance the electron transfer rate, but can also accelerate the oxygen evolution reaction and form a competing reaction with tetracycline degradation, which affects the electrode stability and increases the energy consumption [50,51]. Hence, the experiments for assessing the effect of current density on the tetracycline degradation efficiency were performed by applying current densities in the range of 10~40 mA cm^−2^ at an initial pH of 7 with 50 mg L^−1^ tetracycline and 3.5 g L^−1^ NaCl. As shown in Figure 4, by increasing the current density, the tetracycline degradation was dramatically enhanced; meanwhile, the amount of TOC removal was also increased. The porous Ti/Y_2_O_3_-RuO_2_-TiO_2_@Pt electrode exhibited the highest tetracycline degradation efficiency among the as-prepared electrodes. With the increase in current density, the reaction time of 100% tetracycline degradation was reduced from 15 min at 10 mA cm^−2^ to 5 min at 30 and 40 mA cm^−2^, and the kinetic constant was increased from 0.23 to 1.29 min^−1^. The kinetics of tetracycline degradation fitted well with the pseudo-first-order rate law with increasing current densities. Additionally, the TOC removal also increased from 25.4% to 39.8% as the current density increased from 10 to 40 mA cm^−2^. When the current density increased from 10 to 20 mA cm^−2^, the TOC removal ratio increased by 13.4% after a 20 min reaction time, and when further increasing the current density to 40 mA cm^−2^, the amount of TOC removal increased slowly (Figure S6). As a result, the best energy consumption (EC) for the porous Ti/Y_2_O_3_-RuO_2_-TiO_2_@Pt electrode was 167 kW kg^−1^ TOC at the current density of 20 mA cm^−2^ in the tetracycline degradation process. For the porous Ti/RuO_2_-IrO_2_@Pt and Ti/RuO_2_-TiO_2_@Pt electrode, the tetracycline degradation, kinetic constant, and TOC removal displayed a positive correlation with the current density, and the most efficient result was obtained by using the porous Ti/RuO_2_-IrO_2_@Pt electrode at 40 mA cm^−2^ for a reaction of 60 min.

#### 2.3.3. Effect of Initial Tetracycline Concentration and pH

**Figure 5:**
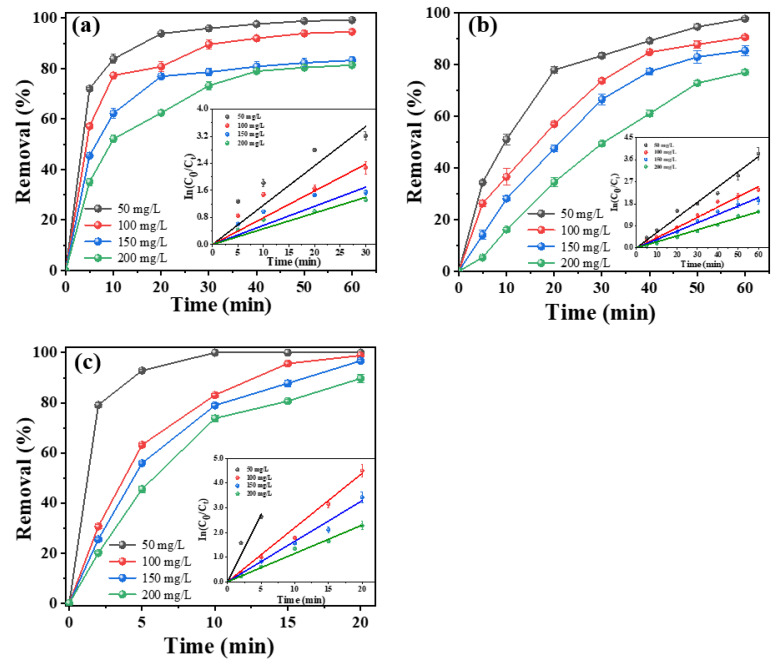
Effect of initial TC concentration on degradation efficiency. (**a**) Porous Ti/RuO_2_-IrO_2_@Pt, (**b**) porous Ti/RuO_2_-TiO_2_@Pt, (**c**) porous Ti/Y_2_O_3_-RuO_2_-TiO_2_@Pt electrodes. Experimental conditions: current density of 20 mA cm^−2^ and electrolyte concentration of 3.5 g L^−1^ NaCI, pH = 7. Inserts show the corresponding kinetics analysis.

**Figure 6:**
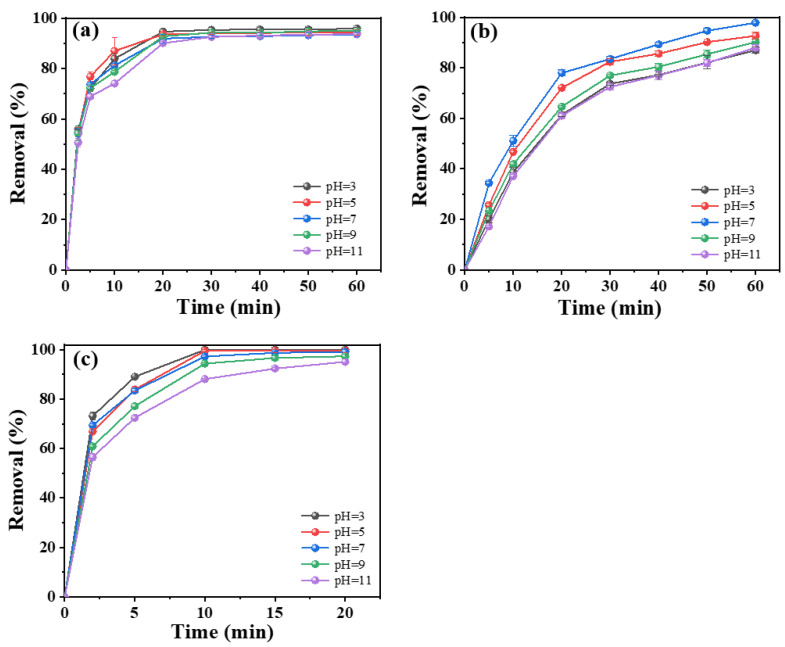
Effect of pH on degradation efficiency. (**a**) Porous Ti/RuO_2_-IrO_2_@Pt, (**b**) porous Ti/RuO_2_-TiO_2_@Pt, (**c**) porous Ti/Y_2_O_3_-RuO_2_-TiO_2_@Pt electrodes. Experimental conditions: current density of 20 mA cm^−2^, TC initial concentration of 50 mg L^−1^, and electrolyte concentration of 3.5 g L^−1^ NaCI. Inserts show the corresponding kinetics analysis.

For practical applications, the initial tetracycline concentration is an important factor that affects the ECO performance. In this work, initial concentrations in the range of 50–200 mg L^−1^ were examined in regard to the degradation of tetracycline. As shown in Figure 5, the removal efficiency decreased with the increase in the tetracycline concentration for all the electrodes. For the porous Ti/Y_2_O_3_-RuO_2_-TiO_2_@Pt electrode, removal rates of tetracycline ranging from 82.9% to 100% were achieved in a 20 min reaction time, which was much higher than that of the other two electrodes. We also observed that porous Ti/Y_2_O_3_-RuO_2_-TiO_2_@Pt electrodes exhibited good electrocatalytic performance at low tetracycline concentration, and the k value was reduced from 0.55 to 0.12 min^−1^. This indicated that the electrocatalytic reaction was probably controlled by the surface adsorption and reaction process, rather than the mass transfer control. The initial pH value was taken into account to evaluate its effect on the electrocatalytic degradation of tetracycline (Figure 6). For all the reaction systems with porous Ti/RuO_2_-TiO_2_@Pt, Ti/RuO_2_-IrO_2_@Pt, and Ti/Y_2_O_3_-RuO_2_-TiO_2_@Pt electrodes, the initial pH showed a non-significant effect on the tetracycline degradation (more than 95% tetracycline removal efficiency), which suggested that the electrodes exhibited good applicability for tetracycline degradation in a wide pH range [52,53]. Commonly, the removal efficiency of tetracycline was faster in acidic conditions than in basic ones. This may be ascribed to the acceleration of •OH production under acidic conditions, and the •OH displayed higher oxidation capability in acidic pH (2.85 V) than in alkaline pH (2.02 V). In addition, for porous Ti/RuO_2_-IrO_2_@Pt and Ti/Y_2_O_3_-RuO_2_-TiO_2_@Pt electrodes, the influence of the initial pH value was slightly higher than that observed for the porous Ti/RuO_2_-TiO_2_@Pt electrode.

### 2.4. Analysis of •OH Production

**Figure 7:**
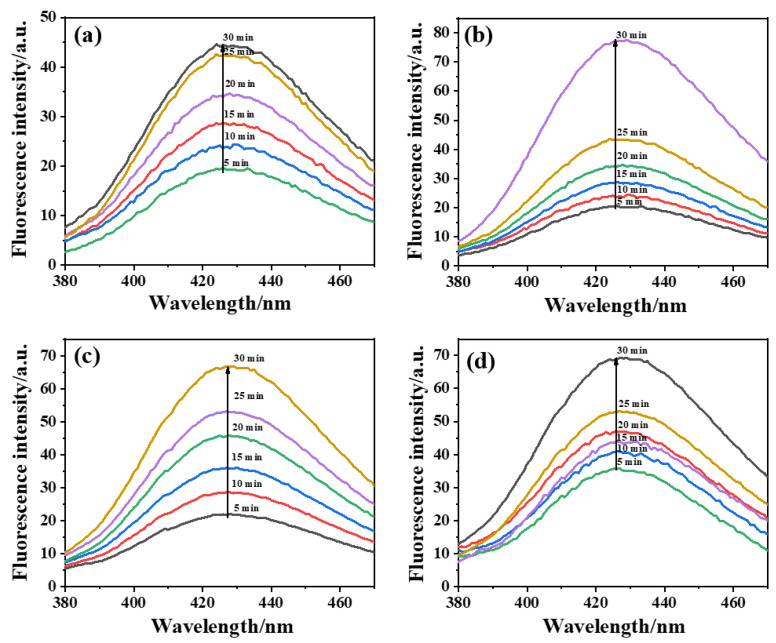
Fluorescence spectra of (**a**) Ti/RuO_2_-IrO_2_, (**b**) porous Ti/RuO_2_-IrO_2_@Pt, (**c**) porous Ti/RuO_2_-TiO_2_@Pt, and (**d**) porous Ti/Y_2_O_3_-RuO_2_-TiO_2_@Pt electrodes. Experimental conditions: electrolysis in 0.5 M terephthalic acid solution for 30 min.

In the ECO process, •OH production was considered the main factor that triggers the degradation of tetracycline [54,55]. Therefore, it was important to monitor the •OH generation during the ECO treatment. Herein, terephthalic acid was used to capture the generated •OH to form a fluorescent byproduct, 2-hydroxy terephthalic acid. As shown in Figure 7, the fluorescence intensity was used to represent the amount of •OH. For all the electrodes, we observed that the fluorescence intensity around 425 nm increased along with the reaction time, which confirmed that •OH was continuously produced on the anode surface. Comparing the fluorescence intensity of all the electrodes indicated that the commercial Ti/RuO_2_-IrO_2_ had the lowest fluorescence intensity among the electrodes, which was probably due to its planar structure and its lower ECSA. Therefore, the porous electrodes had a higher oxidation capability compared with the planar ones.

### 2.5. Evaluation of Different Anodes in Tetracycline Degradation

**Figure 8:**
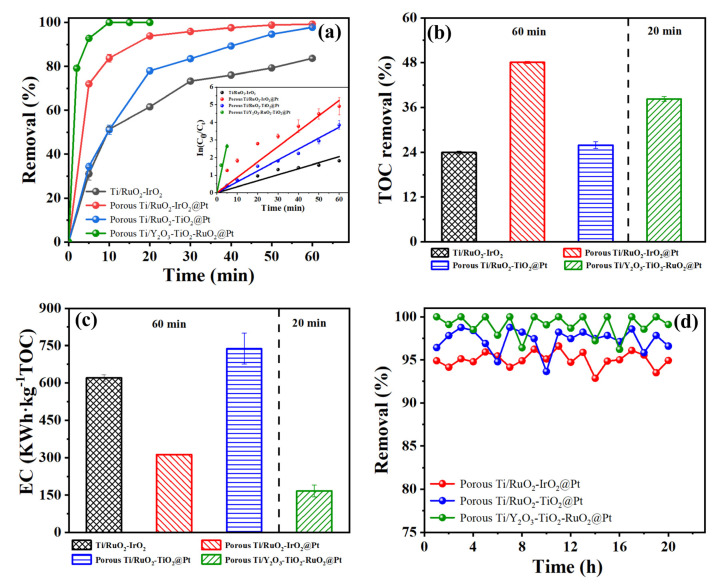
TC degradation performance of Ti/RuO_2_-IrO_2_, porous Ti/RuO_2_-IrO_2_@Pt, porous Ti/RuO_2_-TiO_2_@Pt, and porous Ti/Y_2_O_3_-RuO_2_-TiO_2_@Pt electrodes under optimal conditions. Experimental condition: current density of 20 mA cm^−2^, TC initial concentration of 50 mg L^−1^, and electrolyte concentration of 3.5 g L^−1^ NaCI, pH = 7. (**a**) TC degradation efficiencies and first-order kinetic curves, (**b**) TOC removal, (**c**) TOC energy consumption, (**d**) degradation of TC in twenty consecutive experiments at porous Ti/RuO_2_-IrO_2_@Pt, porous Ti/RuO_2_-TiO_2_@Pt, and porous Ti/Y_2_O_3_-RuO_2_-TiO_2_@Pt electrodes.

Figure 8 depicts the results of our evaluation of the degradation efficiency and energy consumption of tetracycline degradation displayed by the obtained anodes under the same reaction conditions. As shown in Figure 8a, the porous Ti/Y_2_O_3_-RuO_2_-TiO_2_@Pt electrode achieved 100% tetracycline degradation in 20 min corresponding to a k value of 0.55 min^−1^, which was much faster than the other electrodes. Meanwhile, 38.2% TOC removal was also reached after a reaction of 20 min (Figure 8b). For planar Ti/RuO_2_-IrO_2_, with porous Ti/RuO_2_-TiO_2_@Pt electrodes, the TOC removal was only about 24.0% and 25.9% in 60 min, respectively. On the other hand, for porous Ti/RuO_2_-IrO_2_@Pt electrodes, 48.1% TOC removal was achieved in 60 min. Figure 8c shows the energy consumption comparison. The EC results can be expressed in the following order: porous Ti/RuO_2_-TiO_2_@Pt (738 kWh kg^−1^ TOC), planar Ti/RuO_2_-IrO_2_ (621 kWh kg^−1^ TOC), porous Ti/RuO_2_-IrO_2_@Pt (312 kWh kg^−1^ TOC), and porous Ti/Y_2_O_3_-RuO_2_-TiO_2_@Pt (167 kWh kg^−1^ TOC), when comparing all the tested anodes. Their continuous operation was further tested. As shown in Figure 8d, it was observed that all the porous electrodes displayed a stable performance for tetracycline degradation under continuous operation [56,57]. Based on the above-mentioned comparison, it can be concluded that the ECO technology with porous electrodes represents an efficient alternative due to its low energy consumption and high oxidation efficiency.

## 3. Experimental

### 3.1. Chemical Reagents and Materials

H_2_PtCI_6_·6H_2_O, RuCI_3_·3H_2_O, and IrCl_3_·xH_2_O were purchased from Guiyan Platinum Industry Co., Ltd., Guiyang, China. TiCI_3_ and Y(NO_3_)_3_·6H_2_O were purchased from Shanghai Aladdin Biochemical Technology Co., Ltd., Shanghai, China. C_7_H_8_, CTAB, C_6_H_8_O_7_, C_2_H_2_O_4_·2H_2_O, C_2_H_6_O, C_3_H_8_O, and C_3_H_6_O were purchased from Tianjin Tianli Chemical Reagent Co., Ltd., Tianjin, China. Tetracycline hydrochloride (C_22_H_24_N_2_O_8_·HCl) was purchased from Shanghai Maclin Biochemical Technology Co., Ltd., Shanghai, China. A porous titanium sheet with 99.7 % purity and 2 mm thickness was purchased from Baoji Titanium Industry Co., Ltd., Baoji, China. The reagents used were all analytically pure.

### 3.2. Electrode Preparation

**Figure 9:**
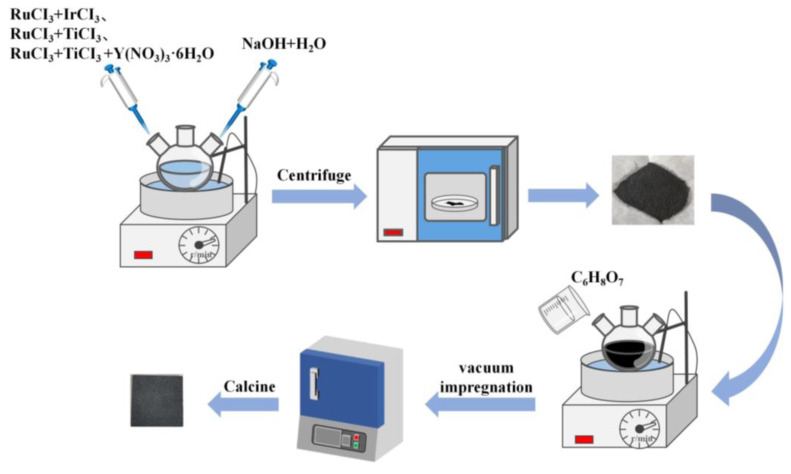
Electrode preparation flow chart.

First, the porous titanium plates were ultrasonically cleaned with deionized water, acetone, and ethanol for 30 min to remove the surface oil. The three nanoparticle powders prepared by means of the microemulsion method (Figure 9) were dissolved in C_3_H_8_O to form a stable and dispersed coating solution. Then, the porous titanium plate was impregnated in the coating solution at vacuum and calcined in the muffle furnace at 350 °C for 15 min. After repeated impregnation and calcination, which was conducted 25 times, porous Ti/RuO_2_-IrO_2_@Pt, porous Ti/RuO_2_-TiO_2_@Pt, and porous Ti/Y_2_O_3_-TiO_2_-RuO_2_@Pt electrodes were finally prepared via calcination at 450 °C for 2 h in a muffle furnace.

### 3.3. Electrode Characterization and Electrochemical Testing

The morphology of the electrodes was analyzed using a scanning electron microscope (SEM, Zeiss Gemini 300, Jena, Germany). We used an X-ray diffractometer (XRD) (D/max2200PC, Tokyo, Japan) with a Cu Kα ray (λ = 0.15406 nm), a scanning voltage of 20 kV, a scanning step of 0.015°, a scanning speed of 1 s/step, and a scanning range of 10°–90° for XRD analysis. Fluorescence spectroscopy (Edinburgh FS5, Livingstone, UK) was performed in the measurement range of 365–535 nm with an excitation wavelength set at 315 nm and an emission wavelength set at 425 nm. The porosity and pore size distribution of porous titanium-based electrodes were analyzed using a mercury piezometer (Micromeritics AutoPore IV 9500, Norcross, GA, USA).

The electrochemical characterization of the composite electrodes was performed using an electrochemical workstation (CHI660E, Shanghai Chenhua Instruments Co., Ltd., Shanghai, China). A three-electrode system was used, with a saturated calomel electrode as the reference electrode, a platinum sheet electrode as the auxiliary electrode, and the prepared porous anodes as the working electrode. Furthermore, 1 mol L^−1^ KOH was used as the electrolyte, and the electrode was subjected to cyclic voltammetry (CV) testing and linear scanning voltammetry (LSV) testing. Finally, the stability of the electrode was evaluated by means of intensive lifetime testing under 1 mol L^−1^ H_2_SO_4_ as the electrolyte and a 2 A cm^−2^ current density.

### 3.4. Tetracycline Electrocatalytic Degradation Evaluation

The concentration of tetracycline hydrochloride was determined by means of ultra-performance liquid chromatography (UHPLC) (waters, Milford, MA, USA). Chromatographic column: C18 (2.1 × 100 mm, 1.7 μm); mobile phase: 35% pure water—65% methanol; flow rate: 0.2 mL/min; UV detector wavelength: 356 nm; injection volume: 10 μL.

The prepared porous titanium composite electrode was used as the anode and the titanium plate was used as the cathode, and a DC regulated power supply was connected to the cathode and anode. For degradation, the distance between the two electrodes was fixed at 2 cm, the area of the electrode immersed in the wastewater was 3 cm^2^, and the degradation was carried out under magnetic stirring and other conditions for 1 h. The effects of different factors (electrolyte type and concentration, current density, initial concentration, pH) on the performance of the electrode degradation of tetracycline were investigated and the optimal conditions were derived. Under optimal conditions, continuous degradation experiments were carried out, with 20 consecutive degradations for a total of 20 h, and samples were taken every hour.

Tetracycline degradation conformed to the pseudo-first-order kinetic model [58], and was calculated by means of Equation (1):

(1) $$\mathrm{In}\left(C_{0}/C_{t}\right)=\mathrm{kt}$$

where C_0_ and C_t_ are the initial concentrations of the pollutant (mg L^−1^) and the concentration with reaction time t (mg L^−1^); K is the pseudo-first-order kinetic rate constant (min^−1^).

The changes in TOC during the reaction were analyzed using a total organic carbon analyzer with the NPOC method. The energy consumption of the metal composite electrode in the electrochemical oxidation and degradation of tetracycline is expressed as kWh kg^−1^ TOC; that is, the electrical energy consumed for each removal of 1 kg TOC [59,60], calculated using Equation (2):

(2) $$EC_{\mathrm{TOC}}\left(\mathrm{kWh}\cdot kg^{-1}\mathrm{TOC}\right)=\frac{1000\mathrm{UIt}}{\Delta \mathrm{TOC}\times V}$$

where U is the voltage (V), I is the current (A), t is the reaction time (h), V is the volume of solution (L), and ∆TOC is the amount of TOC removed from the solution at time t (mg L^−1^).

## 4. Conclusions

In this work, porous anodes (Ti/RuO_2_-TiO_2_@Pt, Ti/RuO_2_-IrO_2_@Pt, and Ti/Y_2_O_3_-RuO_2_-TiO_2_@Pt) were fabricated with a high porosity titanium plate as the substrates. Compared with the planar anode, porous anodes exhibited a larger electrochemical active surface area and a longer service life. The indirect oxidation process triggered by •OH was proven to be responsible for the efficient tetracycline degradation. The porous Ti/Y_2_O_3_-RuO_2_-TiO_2_@Pt anode was found to have the fastest degradation rate of tetracycline and lower energy consumption compared with other anodes with different compositions, which was ascribed to its excellent electrochemical properties. This study thus presents alternatives for the practical application of ECO technology, providing a new option in the form of porous, highly efficient, and long serving life anodes for future industrial application.

## Data Availability

Not available.

## Supplementary Materials

The following supporting information can be downloaded at: https://www.mdpi.com/article/10.3390/molecules28052189/s1, Figure S1: SEM image of porous Ti substrate; Figure S2: Comparison of the piezometric mercury of porous Ti, porous Ti/RuO_2_-IrO_2_@Pt, porous Ti/RuO_2_-TiO_2_@Pt, porous Ti/Y_2_O_3_-RuO_2_-TiO_2_@Pt; Figure S3: Mapping of porous Ti/RuO_2_-IrO_2_@Pt, porous Ti/RuO_2_-TiO_2_@Pt, porous Ti/Y_2_O_3_-RuO_2_-TiO_2_@Pt electrodes (a–h), EDS of (i) porous Ti/RuO_2_-IrO_2_@Pt, (j) porous Ti/RuO_2_-TiO_2_@Pt, (h) porous Ti/Y_2_O_3_-RuO_2_-TiO_2_@Pt electrodes; Figure S4: CV curves of (a) porous Ti/RuO_2_-IrO_2_@Pt, (b) porous Ti/RuO_2_-TiO_2_@Pt, (c) porous Ti/Y_2_O_3_-RuO_2_-TiO_2_@Pt, (d) Ti/RuO_2_-IrO_2_ electrodes at different scanning rates; Figure S5: Effect of electrolyte concentration on TOC and energy consumption of tetracycline; Figure S6: Effect of current density on TOC and energy consumption of tetracycline; Table S1: Pore size parameters of porous Ti-based composite electrodes.

## Sample Availability

Samples of the compounds are available from the authors.

## References

[1] Wang H., Chen T., Chen D., Zou X., Li M., Huang F., Sun F., Wang C., Shu D., Liu H. (2020). Sulfurized oolitic hematite as a heterogeneous Fenton-like catalyst for tetracycline antibiotic degradation. Appl. Catal. B Environ..

[2] Zhang B., He X., Yu C., Liu G., Ma D., Cui C., Yan Q., Zhang Y., Zhang G., Ma J. (2022). Degradation of tetracycline hydrochloride by ultrafine TiO_2_ nanoparticles modified g-C_3_N_4_ heterojunction photocatalyst: Influencing factors, products and mechanism insight. Chin. Chem. Lett..

[3] Wu Z., Tong Z., Xie Y., Sun H., Gong X., Qin P., Liang Y., Yuan X., Zou D., Jiang L. (2022). Efficient degradation of tetracycline by persulfate activation with Fe, Co and O co−doped g−C_3_N_4_: Performance, mechanism and toxicity. Chem. Eng. J..

[4] Cai A., Deng J., Xu M., Zhu T., Zhou S., Li J., Wang G., Li X. (2020). Degradation of tetracycline by UV activated monochloramine process: Kinetics, degradation pathway, DBPs formation and toxicity assessment. Chem. Eng. J..

[5] Wang Y., Rao L., Wang P., Shi Z., Zhang L. (2020). Photocatalytic activity of N-TiO_2_/O-doped N vacancy g-C_3_N_4_ and the intermediates toxicity evaluation under tetracycline hydrochloride and Cr(VI) coexistence environment. Appl. Catal. B Environ..

[6] Shen Q., Wei L., Bibi R., Wang K., Hao D., Zhou J., Li N. (2021). Boosting photocatalytic degradation of tetracycline under visible light over hierarchical carbon nitride microrods with carbon vacancies. J. Hazard. Mater..

[7] Wu Y., Xu Y., Zhang Y., Feng J., Li Y., Lan J., Cheng X. (2022). Fabrication of NiCoP decorated TiO_2_/polypyrrole nanocomposites for the effective photocatalytic degradation of tetracycline. Chin. Chem. Lett..

[8] Zhang Z., Chen Y., Wang P., Wang Z., Zuo C., Chen W., Ao T. (2022). Facile fabrication of N-doped hierarchical porous carbons derived from soft-templated ZIF-8 for enhanced adsorptive removal of tetracycline hydrochloride from water. J. Hazard. Mater..

[9] Jang H.M., Yoo S., Choi Y.K., Park S., Kan E. (2018). Adsorption isotherm, kinetic modeling and mechanism of tetracycline on Pinus taeda-derived activated biochar. Bioresour. Technol..

[10] Xin S., Huo S., Xin Y., Gao M., Wang Y., Liu W., Zhang C., Ma X. (2022). Heterogeneous photo-electro-Fenton degradation of tetracycline through nitrogen/oxygen self-doped porous biochar supported CuFeO_2_ multifunctional cathode catalyst under visible light. Appl. Catal. B Environ..

[11] Zhang D., Zhang K., Hu X., Xue Y., Zhang L., Sun Y. (2021). Ball-milled biochar incorporated polydopamine thin-film composite (PDA/TFC) membrane for high-flux separation of tetracyclic antibiotics from wastewater. Sep. Purif. Technol..

[12] Liu L., Chen Z., Zhang J., Shan D., Wu Y., Bai L., Wang B. (2021). Treatment of industrial dye wastewater and pharmaceutical residue wastewater by advanced oxidation processes and its combination with nanocatalysts: A review. J. Water Process Eng..

[13] Xie W., Shi Y., Wang Y., Zheng Y., Liu H., Hu Q., Wei S., Gu H., Guo Z. (2021). Electrospun iron/cobalt alloy nanoparticles on carbon nanofibers towards exhaustive electrocatalytic degradation of tetracycline in wastewater. Chem. Eng. J..

[14] Ganiyu S.O., van Hullebusch E.D., Cretin M., Esposito G., Oturan M.A. (2015). Coupling of membrane filtration and advanced oxidation processes for removal of pharmaceutical residues: A critical review. Sep. Purif. Technol..

[15] Miklos D.B., Remy C., Jekel M., Linden K.G., Drewes J.E., Hubner U. (2018). Evaluation of advanced oxidation processes for water and wastewater treatment—A critical review. Water Res..

[16] Sires I., Brillas E., Oturan M.A., Rodrigo M.A., Panizza M. (2014). Electrochemical advanced oxidation processes: Today and tomorrow. A review. Environ. Sci. Pollut. Res. Int..

[17] Ammar H.B., Brahim M.B., Abdelhédi R., Samet Y. (2016). Green electrochemical process for metronidazole degradation at BDD anode in aqueous solutions via direct and indirect oxidation. Sep. Purif. Technol..

[18] Kaur R., Kushwaha J.P., Singh N. (2018). Electro-oxidation of Ofloxacin antibiotic by dimensionally stable Ti/RuO_2_ anode: Evaluation and mechanistic approach. Chemosphere.

[19] Yao Y., Jiao L., Yu N., Guo F., Chen X. (2015). Comparison of electrocatalytic characterization of Ti/Sb-SnO_2_ and Ti/F-PbO_2_ electrodes. J. Solid State Electrochem..

[20] Wang Y., Shen C., Zhang M., Zhang B.-T., Yu Y.-G. (2016). The electrochemical degradation of ciprofloxacin using a SnO_2_-Sb/Ti anode: Influencing factors, reaction pathways and energy demand. Chem. Eng. J..

[21] Zeng Y., Zhang S., Yin L., Dai Y. (2022). Electrocatalytic degradation of pesticide micropollutants in water by high energy pulse magnetron sputtered Pt/Ti anode. Chin. Chem. Lett..

[22] Yang W., Tan J., Chen Y., Li Z., Liu F., Long H., Wei Q., Liu L., Ma L., Zhou K. (2022). Relationship between substrate type and BDD electrode structure, performance and antibiotic tetracycline mineralization. J. Alloys Compd..

[23] Yu L., Chen Y., Han W., Sun X., Li J., Wang L. (2016). Preparation of porous TiO_2_-NTs/m-SnO_2_-Sb electrode for electrochemical degradation of benzoic acid. RSC Adv..

[24] Zhang Y., Ding J., Gao Q., Jiang B., Li C., Zhao Q. (2022). Synthesis of low-cost Ti_4_O_7_ membrane electrode for electrooxidation of tetracycline under flow-through conditions: Performance, kinetics and mechanism. Process Saf. Environ. Prot..

[25] Asim S., Zhu Y., Batool A., Hailili R., Luo J., Wang Y., Wang C. (2017). Electrochemical treatment of 2, 4-dichlorophenol using a nanostructured 3D-porous Ti/Sb-SnO_2_-Gr anode: Reaction kinetics, mechanism, and continuous operation. Chemosphere.

[26] Asim S., Zhu Y., Rana M., Yin J., Shah M.W., Li Y., Wang C. (2017). Nanostructured 3D-porous graphene hydrogel based Ti/Sb-SnO_2_-Gr electrode with enhanced electrocatalytic activity. Chemosphere.

[27] Pang D., Liu Y., Song H., Chen D., Zhu W., Liu R., Yang H., Li A., Zhang S. (2021). Trace Ti^3+^- and N-codoped TiO_2_ nanotube array anode for significantly enhanced electrocatalytic degradation of tetracycline and metronidazole. Chem. Eng. J..

[28] Chen M., Pan S., Zhang C., Wang C., Zhang W., Chen Z., Zhao X., Zhao Y. (2020). Electrochemical oxidation of reverse osmosis concentrates using enhanced TiO_2_-NTA/SnO_2_-Sb anodes with/without PbO_2_ layer. Chem. Eng. J..

[29] Lin H., Xiao R., Xie R., Yang L., Tang C., Wang R., Chen J., Lv S., Huang Q. (2021). Defect Engineering on a Ti_4_O_7_ Electrode by Ce^3+^ Doping for the Efficient Electrooxidation of Perfluorooctanesulfonate. Environ. Sci. Technol..

[30] You S., Liu B., Gao Y., Wang Y., Tang C.Y., Huang Y., Ren N. (2016). Monolithic Porous Magnéli-phase Ti_4_O_7_ for Electro-oxidation Treatment of Industrial Wastewater. Electrochim. Acta.

[31] Xu K., Peng J., Chen P., Gu W., Luo Y., Yu P. (2019). Preparation and Characterization of Porous Ti/SnO_2_–Sb_2_O_3_/PbO_2_ Electrodes for the Removal of Chloride Ions in Water. Processes.

[32] Yang D., Tian Z., Song J., Lu T., Qiu G., Kang J., Zhou H., Mao H., Xiao J. (2021). Influences of sintering temperature on pore morphology, porosity, and mechanical behavior of porous Ti. Mater. Res. Express.

[33] Shen J., Chen D., Zhao W., Zhang W.W., Zhou H. (2018). Study on the Preparation and Characterizations of an Improved Porous Ti/TiO_2_/CdS-CNT/C_3_N_4_ Photoelectrode and Photoelectric Catalytic Degradation of Methylene Blue. ChemistrySelect.

[34] Moradi F., Dehghanian C. (2014). Addition of IrO_2_ to RuO_2_+TiO_2_ coated anodes and its effect on electrochemical performance of anodes in acid media. Prog. Nat. Sci. Mater. Int..

[35] Okur M.C., Akyol A., Nayir T.Y., Kara S., Ozturk D., Civas A. (2022). Performance of Ti/RuO_2_-IrO_2_ electrodes and comparison with BDD electrodes in the treatment of textile wastewater by electro-oxidation process. Chem. Eng. Res. Des..

[36] Cheng J., Zhang H., Chen G., Zhang Y. (2009). Study of Ir_x_Ru_1−x_O_2_ oxides as anodic electrocatalysts for solid polymer electrolyte water electrolysis. Electrochim. Acta.

[37] Zhou J., Wang T., Cheng C., Pan F., Zhu Y., Ma H., Niu J. (2022). Ultralong-lifetime Ti/RuO_2_-IrO_2_@Pt anodes with a strong metal-support interaction for efficient electrochemical mineralization of perfluorooctanoic acid. Nanoscale.

[38] Wang L., Liu Y., Pang D., Song H., Zhang S. (2022). Simultaneous electrochemical degradation of tetracycline and metronidazole through a high-efficiency and low-energy-consumption advanced oxidation process. Chemosphere.

[39] Wang Y., Chen M., Wang C., Meng X., Zhang W., Chen Z., Crittenden J. (2019). Electrochemical degradation of methylisothiazolinone by using Ti/SnO_2_-Sb_2_O_3_/α, β-PbO_2_ electrode: Kinetics, energy efficiency, oxidation mechanism and degradation pathway. Chem. Eng. J..

[40] Zhi D., Zhang J., Wang J., Luo L., Zhou Y., Zhou Y. (2020). Electrochemical treatments of coking wastewater and coal gasification wastewater with Ti/Ti_4_O_7_ and Ti/RuO_2_-IrO_2_ anodes. J Environ. Manag..

[41] Sheng S., Ye K., Gao Y., Zhu K., Yan J., Wang G., Cao D. (2021). Simultaneously boosting hydrogen production and ethanol upgrading using a highly-efficient hollow needle-like copper cobalt sulfide as a bifunctional electrocatalyst. J. Colloid. Interface Sci..

[42] Chen B., Liu J., Wang S., Huang H., He Y., Guo Z. (2021). Preparation and electrochemical properties of a novel porous Ti/Sn-Sb-RuO_x_/β-PbO_2_/MnO_2_ anode for zinc electrowinning. RSC Adv..

[43] Xie X., Chang L., Chen B., Li J., Huang H., Guo Z., He Y. (2021). Effects of coating precursor states on performance of titanium-based metal oxide coating anode for Mn electrowinning. Electrochim. Acta.

[44] Chen J., Xia Y., Dai Q. (2015). Electrochemical degradation of chloramphenicol with a novel Al doped PbO_2_ electrode: Performance, kinetics and degradation mechanism. Electrochim. Acta.

[45] Li D., Tang J., Zhou X., Li J., Sun X., Shen J., Wang L., Han W. (2016). Electrochemical degradation of pyridine by Ti/SnO_2_–Sb tubular porous electrode. Chemosphere.

[46] Zhou C., Wang Y., Tang S., Wang Y., Yu H., Niu J. (2021). Insights into the electrochemical degradation of triclosan from human urine: Kinetics, mechanism and toxicity. Chemosphere.

[47] Xu J., Liu Y., Li D., Li L., Zhang Y., Chen S., Wu Q., Wang P., Zhang C., Sun J. (2022). Insights into the electrooxidation of florfenicol by a highly active La-doped Ti_4_O_7_ anode. Sep. Purif. Technol..

[48] Wu J., Zhu K., Xu H., Yan W. (2019). Electrochemical oxidation of rhodamine B by PbO_2_/Sb-SnO_2_/TiO_2_ nanotube arrays electrode. Chin. J. Catal..

[49] Wang C., Yin L., Xu Z., Niu J., Hou L.-A. (2017). Electrochemical degradation of enrofloxacin by lead dioxide anode: Kinetics, mechanism and toxicity evaluation. Chem. Eng. J..

[50] Chen S., He P., Wang X., Xiao F., Zhou P., He Q., Jia L., Dong F., Zhang H., Jia B. (2021). Co/Sm-modified Ti/PbO_2_ anode for atrazine degradation: Effective electrocatalytic performance and degradation mechanism. Chemosphere.

[51] Song S., Fan J., He Z., Zhan L., Liu Z., Chen J., Xu X. (2010). Electrochemical degradation of azo dye C.I. Reactive Red 195 by anodic oxidation on Ti/SnO_2_–Sb/PbO_2_ electrodes. Electrochim. Acta.

[52] Li X., Li X., Yang W., Chen X., Li W., Luo B., Wang K. (2014). Preparation of 3D PbO_2_ nanospheres@SnO_2_ nanowires/Ti Electrode and Its Application in Methyl Orange Degradation. Electrochim. Acta.

[53] Dai Q., Zhou J., Meng X., Feng D., Wu C., Chen J. (2016). Electrochemical oxidation of cinnamic acid with Mo modified PbO_2_ electrode: Electrode characterization, kinetics and degradation pathway. Chem. Eng. J..

[54] Zhao W., Xing J., Chen D., Bai Z., Xia Y. (2015). Study on the performance of an improved Ti/SnO_2_–Sb_2_O_3_/PbO_2_ based on porous titanium substrate compared with planar titanium substrate. RSC Adv..

[55] Ishibashi K.-i., Fujishima A., Watanabe T., Hashimoto K. (2000). Detection of active oxidative species in TiO_2_ photocatalysis using the fluorescence technique. Electrochem. Commun..

[56] Bai C., Yang G., Zhang S., Deng S., Zhang Y., Chen C., He J., Xu M., Long L. (2021). A synergistic system of electrocatalytic-anode/α-MnO_2_/peroxymonosulfate for removing combined pollution of tetracycline and Cr(VI). Chem. Eng. J..

[57] Xin S., Ma B., Liu G., Ma X., Zhang C., Ma X., Gao M., Xin Y. (2021). Enhanced heterogeneous photo-Fenton-like degradation of tetracycline over CuFeO_2_/biochar catalyst through accelerating electron transfer under visible light. J Environ. Manag..

[58] Wang P., Yuan Q. (2021). Photocatalytic degradation of tetracyclines in liquid digestate: Optimization, kinetics and correlation studies. Chem. Eng. J..

[59] Bugueno-Carrasco S., Monteil H., Toledo-Neira C., Sandoval M.A., Thiam A., Salazar R. (2021). Elimination of pharmaceutical pollutants by solar photoelectro-Fenton process in a pilot plant. Environ. Sci. Pollut. Res. Int..

[60] Steter J.R., Brillas E., Sirés I. (2018). Solar photoelectro-Fenton treatment of a mixture of parabens spiked into secondary treated wastewater effluent at low input current. Appl. Catal. B Environ..

