# Supplementary material for: Preparation of Porous Ti/RuO2-IrO2@Pt, Ti/RuO2-TiO2@Pt and Ti/Y2O3-RuO2-TiO2@Pt Anodes for Efficient Electrocatalytic Decomposition of Tetracycline
Source: Molecules. 2023 Feb 27;28(5):2189. doi: 10.3390/molecules28052189 (PMC10004508; doi:10.3390/molecules28052189)
Supplement: Supplementary file 1 [file molecules-28-02189-s001.zip › molecules-2219382-supplementary.pdf]

Supporting Materials for

Preparation of porous Ti/RuO_2-IrO_2@Pt, Ti/RuO_2-TiO_2@Pt and Ti/Y_2O_3-RuO_2-TiO_2@Pt Anodes for Efficient Electrocatalytic decomposition of tetracycline

Yunqing Zhu[a*], Bingqing Li[a], Yongming Wang[a], Tian Wang[a], Junfeng Niu[a]

[a] School of Environmental Science and Engineering, Shaanxi University of Science and Technology, Xian 710021, China

Corresponding author:

* Yunqing Zhu

Email address: zhuyunqing@sust.edu.cn

Address: School of Environmental Science and Engineering, Shaanxi University of Science and Technology, Weiyang University Park, Xian 710021, China

Text S1. Electrode preparation

Nanoparticles were prepared by microemulsion method

Preparation of Pt-Ru-Ir nanoparticles: Firstly, 4.044g CTAB was added to 150 ml C_7H_8 under intense mechanical agitation. After the mixed suspension was obtained, H_2PtCI_6▪$6H_2O$ precursor solution was added to the suspension of the previous step by drop and stirred overnight. Then, the solution containing $RuCI_3$▪$3H_2O$ and $IrCl_3 \cdot xH_2O$ was added to the solution prepared in the previous step. After stirring for 30min, 0.223g NaOH solution was added, and the mixture was evenly stirred. Finally, platinum-Ruthenium-iridium nanoparticles were obtained after centrifugation, alcohol washing, and drying in an oven at 100 °C overnight.

Preparation of Pt-Ru-Ti nanoparticles: Firstly, 4.044g CTAB was added to 150 ml C_7H_8 under intense mechanical agitation. After the mixed suspension was obtained, H_2PtCI_6▪$6H_2O$ precursor solution was added to the suspension of the previous step by drop and stirred overnight. Then, the solution containing $RuCI_3$▪$3H_2O$ and $TiCI_3$ was added to the solution prepared in the previous step. After stirring for 30min, 0.223g NaOH solution was added, and the mixture was evenly stirred. Finally, platinum-Ruthenium-iridium nanoparticles were obtained after centrifugation, alcohol washing, and drying in an oven at 100 °C overnight.

Preparation of Pt-Ru-Ti-Y nanoparticles: Firstly, 4.044g CTAB was added to 150 ml C_7H_8 under intense mechanical agitation. After the mixed suspension was obtained, H_2PtCI_6▪$6H_2O$ precursor solution was added to the suspension of the previous step by drop and stirred overnight. Then, the solution containing $RuCI_3$▪$3H_2O$、$TiCI_3$ and $Y(NO_3)_3$ • $6H_2O$ was added to the solution prepared in the previous step. After stirring for 30min, 0.223g NaOH solution was added, and the mixture was evenly stirred. Finally, platinum-Ruthenium-iridium nanoparticles were obtained after centrifugation, alcohol washing, and drying in an oven at 100 °C overnight.

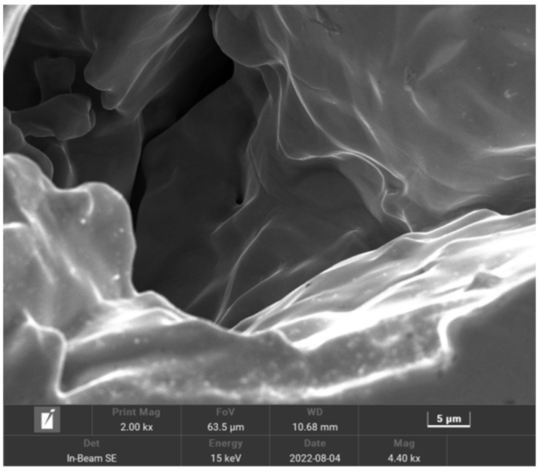

Figure S1. SEM image of porous Ti substrate

Table S1

Pore size parameters of porous Ti-based composite electrodes

Electrode	Average pore size（μm）	Total hole capacity（mL/g）	Total hole area（m^2/g）
Porous Ti	24.47	0.082	0.013
Porous Ti/RuO_2-IrO_2@Pt	10.89	0.093	0.034
Porous Ti/RuO_2-TiO_2@Pt	14.84	0.092	0.025
Porous Ti/Y_2O_3-RuO_2-TiO_2@Pt	12.64	0.09	0.028

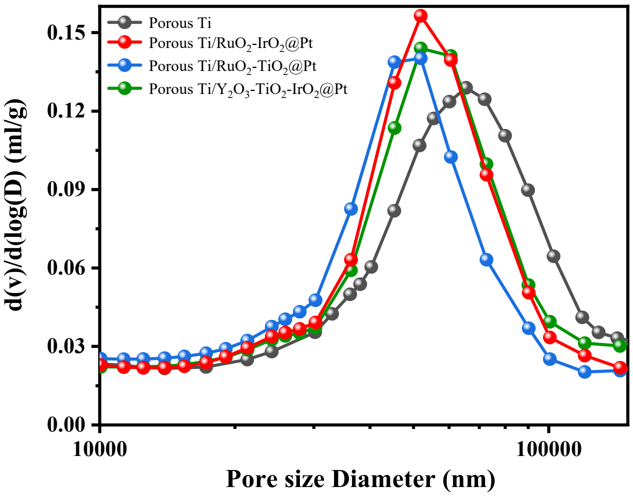

Figure S2. Comparison of the piezometric mercury of porous Ti,porous Ti/RuO_2-IrO_2@Pt, porous Ti/RuO_2-TiO_2@Pt, porous Ti/Y_2O_3-RuO_2-TiO_2@Pt.

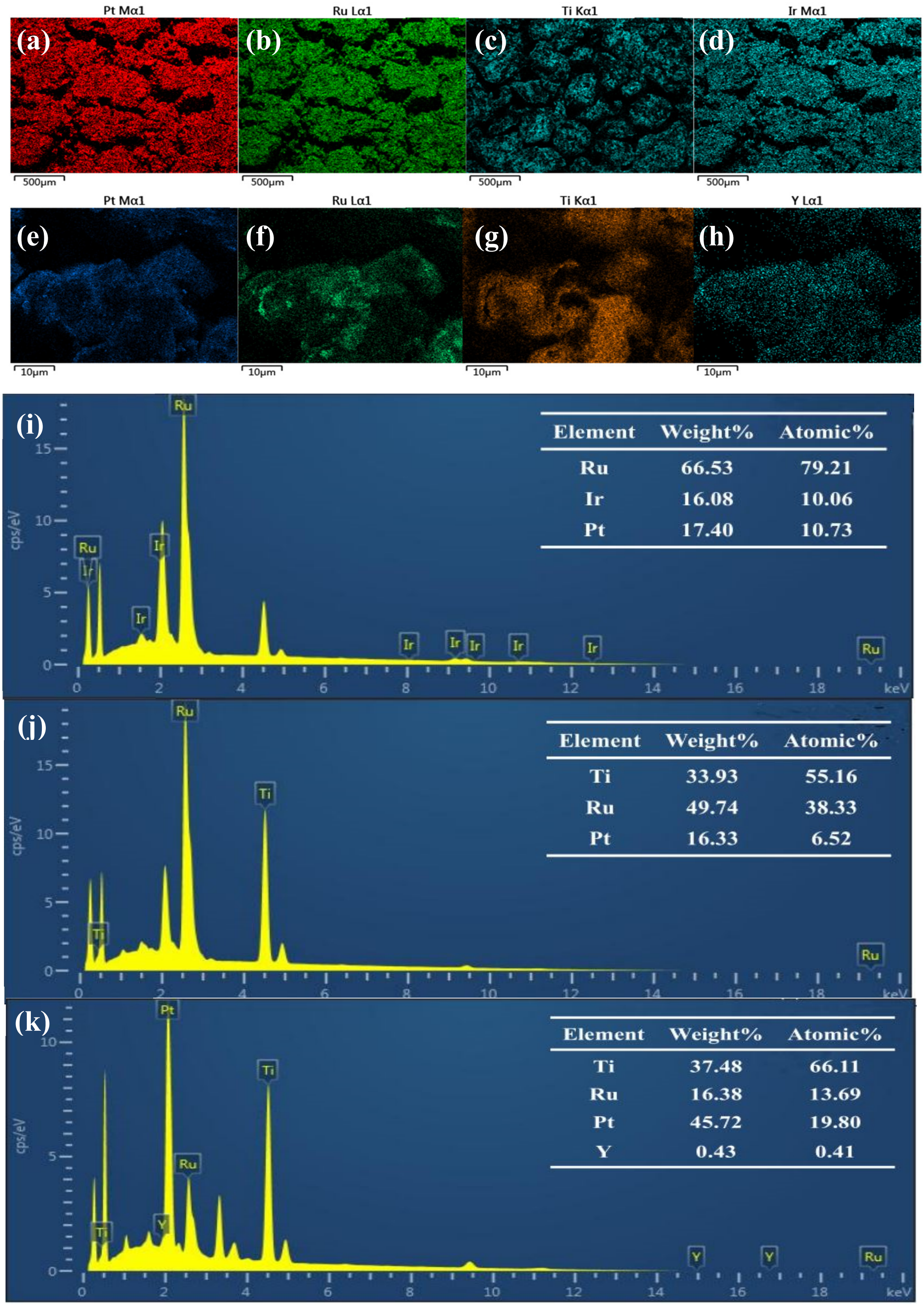

Figure S3. Mapping of porous Ti/RuO_2-IrO_2@Pt, porous Ti/RuO_2-TiO_2@Pt , porous Ti/Y_2O_3-RuO_2-TiO_2@Pt electrodes(a-h), EDS of (i) porous Ti/RuO_2-IrO_2@Pt,(j) porous Ti/RuO_2-TiO_2@Pt,(h) porous Ti/Y_2O_3-RuO_2-TiO_2@Pt electrodes.

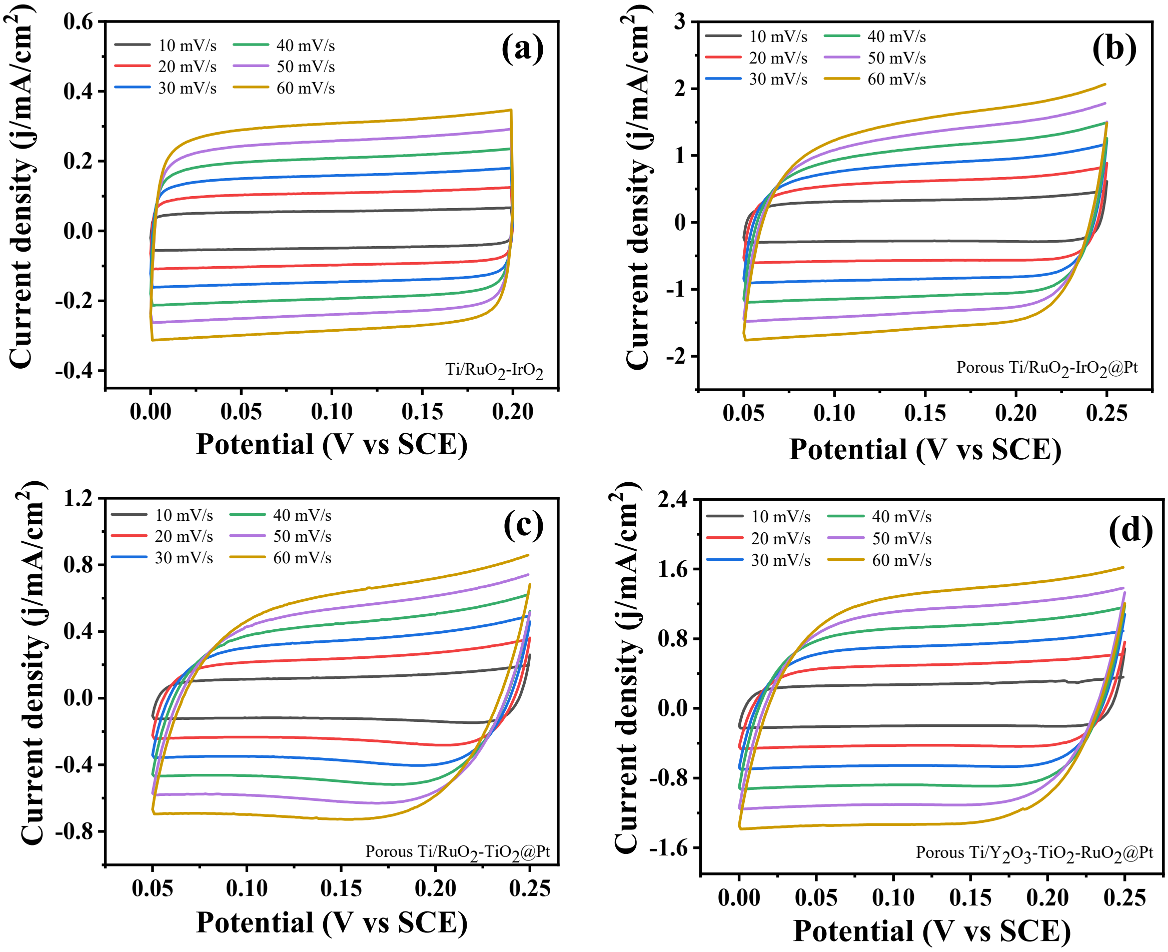

Figure S4. CV curves of (a) porous Ti/RuO_2-IrO_2@Pt,(b) porous Ti/RuO_2-TiO_2@Pt,(c) porous Ti/Y_2O_3-RuO_2-TiO_2@Pt,(d) Ti/RuO_2-IrO_2 electrodes at different scanning rates.

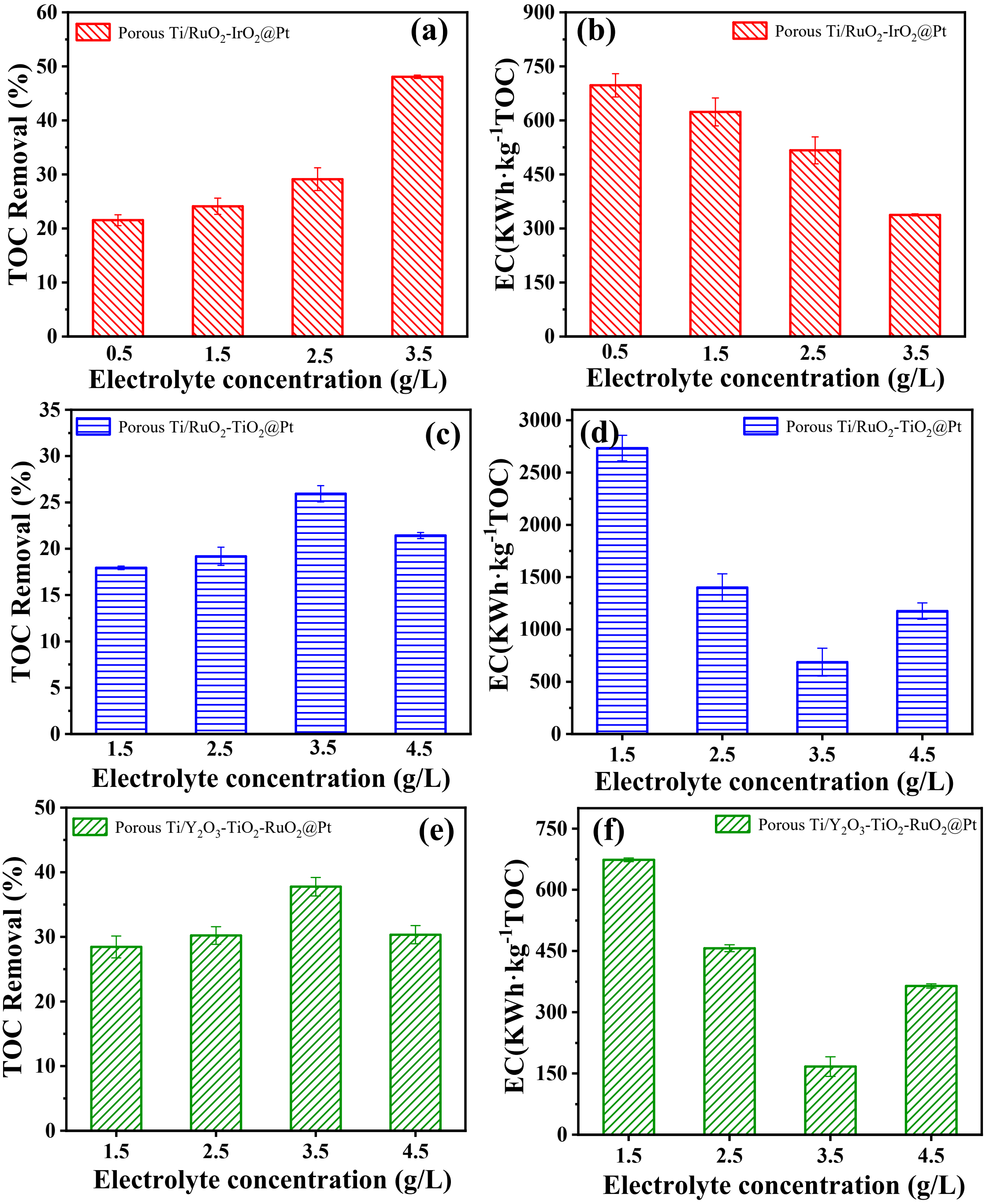

Figure S5. Effect of electrolyte concentration on TOC and energy consumption of tetracycline.(a,b) porous Ti/RuO_2-IrO_2@Pt, (c,d) porous Ti/RuO_2-TiO_2@Pt, (e,f) porous Ti/Y_2O_3-RuO_2-TiO_2@Pt electrodes.

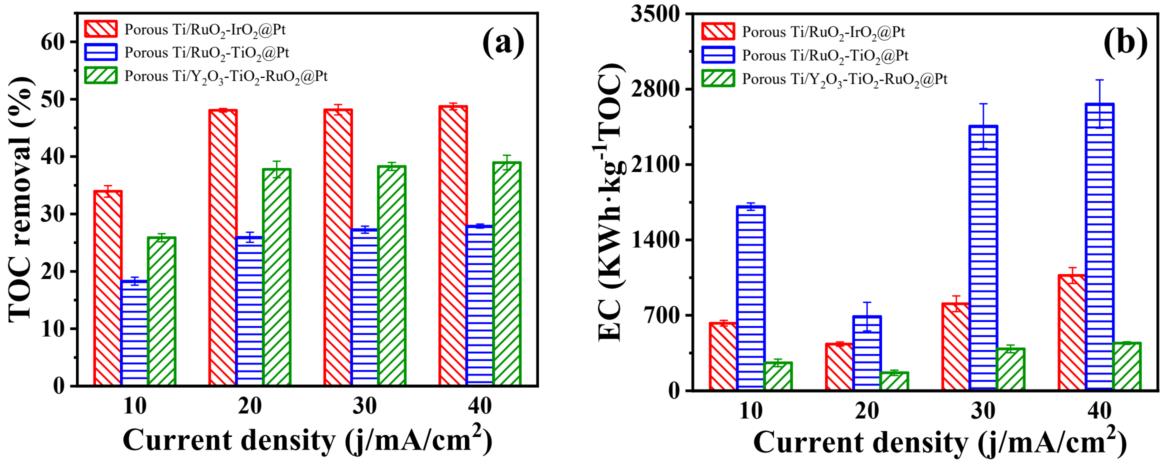

Figure S6. Effect of current density on TOC and energy consumption of tetracycline. (a) TOC removal. (b) TOC energy consumption.

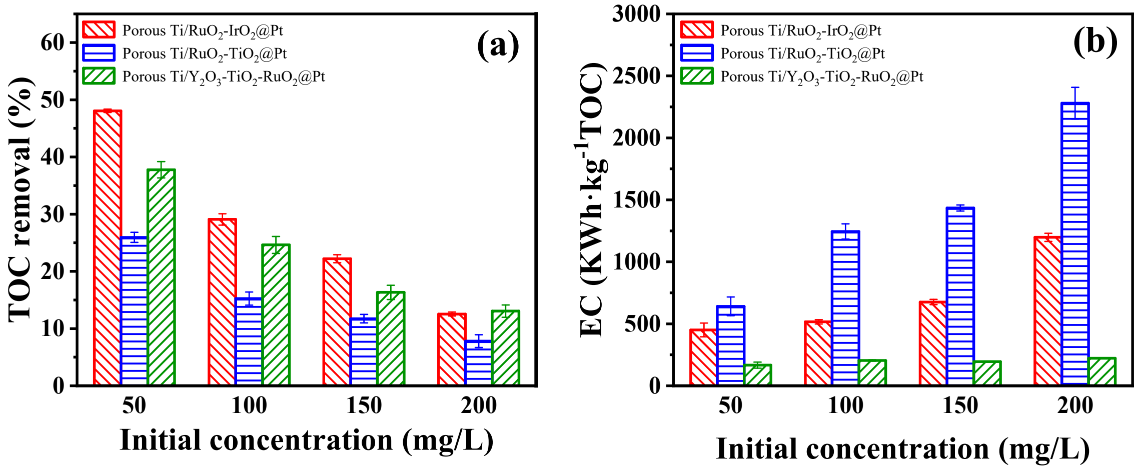

Figure S7. Effect of initial concentration on TOC and energy consumption of tetracycline. (a) TOC removal. (b) TOC energy consumption.

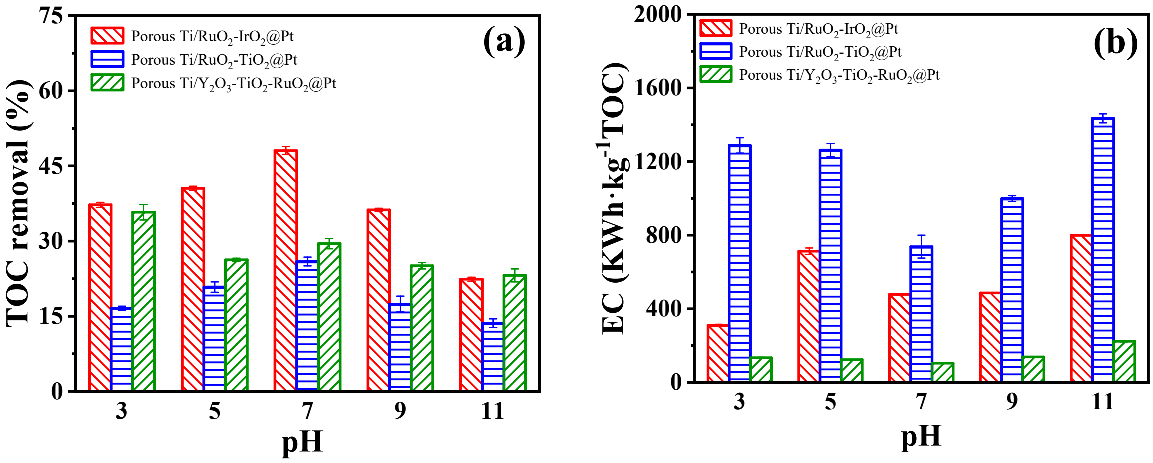

Figure S8. Effect of pH on TOC and energy consumption of tetracycline. (a) TOC removal (b) TOC energy consumption

Table S2

Rate constants of pseudo-first-order kinetic models of tetracycline under different parameters

Parameters			k（min^{-1}）	R^2
electrolyte type	Porous Ti/RuO_2-IrO_2@Pt	NaCI	0.09	0.99
		Na_2SO_4	0.01	0.99
	Porous Ti/RuO_2-TiO_2@Pt	NaCI	0.06	0.99
		Na_2SO_4	0.02	0.99
	Porous Ti/Y_2O_3-RuO_2-TiO_2@Pt	NaCI	0.55	0.98
		Na_2SO_4	0.02	0.95
electrolyte concentration (g/L)	Porous Ti/RuO_2-IrO_2@Pt	0.5	0.04	0.95
		1.5	0.06	0.93
		2.5	0.07	0.94
		3.5	0.09	0.99
	Porous Ti/RuO_2-TiO_2@Pt	1.5	0.03	0.99
		2.5	0.04	0.99
		3.5	0.06	0.99
		4.5	0.05	0.98
	Porous Ti/Y_2O_3-RuO_2-TiO_2@Pt	1.5	0.32	0.99
		2.5	0.48	0.99
		3.5	0.55	0.98
		4.5	0.41	0.99
current density (mA/cm^2)	Porous Ti/RuO_2-IrO_2@Pt	10	0.04	0.99
		20	0.11	0.96
		30	0.12	0.96
		40	0.13	0.94
	Porous Ti/RuO_2-TiO_2@Pt	10	0.03	0.99
		20	0.06	0.99
		30	0.06	0.99
		40	0.08	0.99
	Porous Ti/Y_2O_3-RuO_2-TiO_2@Pt	10	0.23	0.99
		20	0.55	0.98
		30	1.24	0.98
		40	1.29	0.99
initial concentration (mg/L)	Porous Ti/RuO_2-IrO_2@Pt	50	0.12	0.96
		100	0.08	0.98
		150	0.06	0.95
		200	0.05	0.98
	Porous Ti/RuO_2-TiO_2@Pt	50	0.06	0.99
		100	0.04	0.99
		150	0.03	0.99
		200	0.02	0.99
	Porous Ti/Y_2O_3-RuO_2-TiO_2@Pt	50	0.55	0.98
		100	0.22	0.99
		150	0.16	0.99
		200	0.11	0.99

Table S3

Rate constants of pseudo-first-order kinetic models of tetracycline under optimal parameters

Parameters	k（min^{-1}）	R^2
Ti/RuO_2-IrO_2	0.03	0.96
Porous Ti/RuO_2-IrO_2@Pt	0.09	0.99
Porous Ti/RuO_2-TiO_2@Pt	0.06	0.99
Porous Ti/Y_2O_3-RuO_2-TiO_2@Pt	0.55	0.98
